# Effect of Storage Temperature on Storage Life and Sensory Attributes of Packaged Mustard Microgreens

**DOI:** 10.3390/life13020393

**Published:** 2023-01-31

**Authors:** Nayani N. Dayarathna, Nalin S. Gama-Arachchige, Jilushi W. Damunupola, Zhenlei Xiao, Ashoka Gamage, Othmane Merah, Terrence Madhujith

**Affiliations:** 1 Department of Botany, Faculty of Science, University of Peradeniya, Peradeniya 20400, Sri Lanka; 2 Postgraduate Institute of Science, University of Peradeniya, Peradeniya 20400, Sri Lanka; 3 Department of Culinary Science and Product Development, College of Food Innovation & Technology, Johnson & Wales University, Providence, RI 02905, USA; 4 Chemical and Process Engineering, Faculty of Engineering, University of Peradeniya, Peradeniya 20400, Sri Lanka; 5 Laboratoire de Chimie Agro-Industrielle (LCA), Université de Toulouse, INRAe, INPT, 31030 Toulouse, France; 6 Département Génie Biologique, Université Paul Sabatier, IUT A, 32000 Auch, France; 7 Department of Food Science and Technology, Faculty of Agriculture, University of Peradeniya, Peradeniya 20400, Sri Lanka

**Keywords:** microgreens, postharvest quality, sensory attributes, storage temperature

## Abstract

Short shelf life limits the commercial value of mustard microgreens. The present study was conducted to evaluate the effects of different storage temperatures on postharvest quality and sensory attributes of mustard microgreens to identify the optimum storage temperature. Mustard microgreens were stored at 5, 10, 15, 20, and 25 °C in 150 µm polyethylene bags. Samples were drawn at 0, 1, 2, 4, 7, 10, and 14 days and tested for changes in total chlorophyll content, tissue electrolyte leakage, weight loss, antioxidant activity, and sensory attributes. Storage temperature significantly (*p* < 0.05) affected the product quality, shelf life, and sensory quality. When stored at 5 °C, mustard microgreens showed no significant changes in antioxidant activity or tissue electrolyte leakage and minimal change in other parameters and maintained good overall sensory quality for 14 days. Samples stored at 10 and 15 °C retained good overall sensory quality for 4 and 2 days, respectively. When stored at 20 and 25 °C, microgreens deteriorated beyond consumption within one day. A storage temperature of 5 °C in 150 µm polythene bags can preserve high postharvest quality and sensory attributes for 14 days.

## 1. Introduction

A healthy diet protects humans against diet-related non-communicable diseases and malnutrition [1]. Microgreens are a good addition to a healthy diet as they are rich in bioactives such as vitamins, antioxidants, macro- and microelements, and fiber [2,3,4]. Brassicaceous plants are rich in carotenoids (lutein, zeaxanthin, and β-carotene), polyphenol, glucosinolates, tocopherols, and ascorbic acid [5,6,7,8]. Brassicaceous microgreens contain higher levels of bioactive compounds than their mature counterparts and belong to the group of functional foods which provide health promoting or disease preventing properties rather than their nutritional values [2,9,10]. Mustard (*Brassica juncea* (L.) Czern.) is a popular brassicaceous plant grown as a microgreen. Mustard microgreens are rich in bio-accessible antioxidants (β-carotene, sulphoraphane, and cyanidin-3-glucose) and minerals (K, Ca, Mg, Fe, and Zn), making them a good functional food [11]. Red cabbage microgreen supplementation on rats fed with a high-fat diet resulted in attenuated high-fat-diet-induced weight gain, lower LDL levels, reduced hepatic cholesterol ester and triacylglycerol levels, and reduced expression of inflammatory cytokines in the liver [12].

Microgreens have quick production cycles of two to three weeks and require very little space in greenhouse production [13]. The shelf life of microgreens at ambient conditions is 3–5 days [14]. Industrial production and sales of microgreens are, however, limited due to their short shelf life and the rapid deterioration of the quality of the product [15,16]. Microgreens senesce quickly after harvesting, due to the premature interruption of plants at an early stage [17]. The postharvest shelf life of microgreens depends on many factors such as storage temperature, relative humidity, packaging film type, and initial microbial load [18]. The shelf life of microgreens must be extended in order to improve the marketability of this highly perishable commodity [19].

Storage temperature affects the postharvest quality and storage behavior of fresh produce. A reduction in storage temperature by 10 °C can slow down the metabolic activity of fruits and vegetables by a factor of two to three [20]. In general, low-temperature storage reduces quality loss and extends shelf life by depressing the rates of respiration, tissue senescence, and the activities of microorganisms which are capable of causing the spoilage of the products. However, too low a temperature can cause chilling injuries in postharvest products [21]. Therefore, the selection of an optimum storage temperature is crucial as it varies depending on the product [22].

Factors such as knowledge, cost, familiarity with microgreens, availability, freshness, and shelf life influence the purchase of microgreens by consumers [23]. Modified atmospheric packaging, post- and pre-harvest calcium treatments, pre-harvest aloe gel treatments, and post-harvest organic acid treatments are effective in increasing the shelf life of microgreens [2,24,25,26]. However, any additional step in the production line will incur more costs. Therefore, the use of a low temperature seems to be more cost effective as supermarkets are equipped with low-temperature cabinets for fresh produce.

Storage at low temperatures (1–5 °C) has been recommended for some microgreen varieties [2,16,22]. However, no information is available on the effects of storage temperature and time on postharvest quality and sensory attributes of mustard microgreens. Therefore, the objective of this study was to determine the effects of storage temperatures on weight loss, total chlorophyll content, tissue electrolyte leakage, antioxidant activity, and sensorial attributes of mustard microgreens to optimize postharvest shelf life and reduce quality loss.

## 2. Materials and Methods

### 2.1. Sample Preparation and Storage

Mustard seeds (*Brassica juncea* (L.) Czern.) were purchased from a local market in Kandy, Sri Lanka. Seeds were grown in an autoclaved medium (90 min in 120 °C, 120 Pa) of compost and coco peat 1:1 (*v*/*v*) in perforated plastic trays. The trays were kept in a plant house under indirect sunlight. When mustard microgreens reached approx. 5 cm in height, they were harvested by cutting stems just above the soil layer. Microgreens without any defects were selected and washed with water to remove potting material and were allowed to drain at room temperature for 15 min.

Fifteen grams each of freshly harvested mustard microgreens was packed in 160 polyethylene bags (20 × 20 cm, 150 µm gauge) and sealed. Thirty-two of those bags were stored at 5, 10, 15, 20, and 25 °C each. For 5 and 10 °C, refrigerators were used (Super General, SGR-275, Dubai, United Arab Emirates) and for 15, 20, and 25 °C, incubators (Hinotek, MGC-450 BP, Ningbo, China) were used. These samples were used in the analyses of weight loss, total chlorophyl content, and tissue electrolyte leakage and in sensory evaluation experiments.

Mustard microgreens used in the DPPH radical scavenging antioxidant activity were stored in a separate set of bags. Fifty grams of freshly harvested mustard microgreens was packed in 30 polyethylene bags (20 × 20 cm, 150 µm gauge) and sealed. Six of those bags were stored at 5, 10, 15, 20, and 25 °C each, under the same storage conditions mentioned above.

### 2.2. Postharvest Quality Assessments

Four mustard microgreen bags were drawn at 4, 7, 10, and 14 days each from samples stored at 5 and 10 °C; 4 and 7 days from samples stored at 15 °C; and 1 day from samples stored at 20 and 25 °C, which were used in weight loss, total chlorophyl content, and tissue electrolyte leakage analyses. Four replicates (one from each bag) per one temperature treatment were used in each calculation of weight loss, total chlorophyl content, and tissue electrolyte leakage on each evaluation day. Four bags, each with 15 g of freshly harvested microgreens on day 0, were used as the control. Weight loss, total chlorophyll content, and tissue electrolyte leakage experiments were repeated twice. Thus, the total number of replicates per temperature treatment per day was eight.

#### 2.2.1. Analysis of Weight Loss

Weight loss of the mustard microgreens was determined following the procedure described by Xiao et al. [16]. Mustard microgreen samples were weighed (without the bag) at the different storage periods mentioned above. The results were expressed as a percentage of weight loss on a fresh weight basis.

$$\mathrm{Weight}\;\mathrm{loss}\;\%=\frac{\left\{initial\;fresh\;weight\;(g)-Final\;fresh\;weight\;\left(g\right)\right\}\times 100}{Initial\;fresh\;weight\;\left(g\right)}$$

#### 2.2.2. Determination of Total Chlorophyll Content

Total chlorophyll content was determined spectrophotometrically using the method described by Xiao et al. [16] with minor modifications. One gram of cotyledonary leaves without the stems was excised and macerated with 10 mL of 80% acetone (Sigma-Aldrich, St. Louis, MO, USA) using a mortar and pestle. The resulting suspension was transferred into 50 mL centrifuge tubes and centrifuged at 4500 rpm for 5 min (Measuring and Scientific Equipment (MSE 848335, South London, UK). The supernatant was decanted into a volumetric flask, and the resulting pellet was again extracted with 5 mL of 80% acetone at 3000 rpm for 5 min. This procedure was repeated until the filter cake became colorless. The filtrate was diluted with 80% acetone up to 25 mL, and absorbance was measured at 710, 663, and 646 nm using a UV-visible spectrophotometer (UV-Visible spectrophotometer, Spec M02, Spectronic Camspec Ltd., Garforth, UK). The total chlorophyll content (TCC) was calculated at the different storage periods mentioned above.

$$\mathrm{TCC}=\left\{\left(A_{646}-A_{710}\right)\times 0.01732+\left(A_{663}-A_{710}\right)\times 0.00718\times D\times \frac{1000}{FW}\right\}\;$$

TCC = total chlorophyll content (µg/g FW), A_646_ = absorbance at 646 nm, A_663_ = absorbance at 663 nm, A_710_ = absorbance at 710 nm, FW = fresh weight (g), D = dilution volume (mL).

#### 2.2.3. Analysis of Tissue Electrolyte Leakage

Tissue electrolyte leakage was measured following a procedure described by Fan & Sokorai [27] and Xiao et al. [12]. Five grams of mustard microgreens was submerged in 150 mL of deionized water and shaken for 30 min at 20 rpm in a mechanical shaker (Gallen Kamp—IC 8804, Cambridge, UK). The electrical conductivity of the solution was measured at room temperature (~27 °C) using a conductivity meter (Okton—CON 410, Vernon Hills, IL 60061, USA). Total electrical conductivity was measured at the different storage periods mentioned above, after freezing the samples at −20 °C in a freezer (Super General—SGR275, Dubai, United Arab Emirates) for 24 h and subsequently thawing at room temperature. Tissue electrolyte leakage was expressed as a percentage of the total electrical conductivity.

$$\mathrm{Electrolyte}\;\mathrm{leakage}\;(\%)=\frac{\mathrm{Initial}\;\mathrm{electrolyte}\;\mathrm{of}\;\mathrm{the}\;\mathrm{solution}\;(µs)}{\mathrm{Total}\;\mathrm{electrolyte}\;\mathrm{of}\;\mathrm{the}\;\mathrm{solution}\;(µs)}\times 100\;$$

#### 2.2.4. Determination of DPPH Radical Scavenging Antioxidant Activity

##### Preparation of Plant Extracts

Plant extracts were prepared following the procedure described by Sultana et al. [28]. Three bags each (replicates) were drawn on days 7 and 14 from samples stored at 5 and 10 °C; day 7 from samples stored at 15 °C; and day 1 from samples stored at 20 and 25 °C. Three bags, each with 50 g of freshly harvested microgreens on day 0, were used as a control. Samples were oven dried at <35 °C for 24 h, ground, sieved (0.5 mm), and extracted with 100% methanol (Sigma-Aldrich, St. Louis, MO, USA) on a mechanical shaker (2 days) and evaporated to a thick residue. The residue was dissolved in 100% methanol to prepare a 2000 ppm stock solution.

##### DPPH Radical Scavenging Activity

The DPPH radical scavenging assay was carried out according to the procedure described by Tailor & Goyal [29] with minor modifications. A dilution series of plant extract concentrations (62.5, 125, 250, 500, and 1000 ppm) in 100% methanol (Sigma-Aldrich, St. Louis, MO, USA) and 0.3 mmol DPPH solution (Sigma-Aldrich, St. Louis, MO, USA) in methanol were prepared. To 3 mL of each plant extract in methanol, 1.2 mL of DPPH solution was added, and the mixture was shaken vigorously and allowed to stand for 30 min in the dark at room temperature. Absorbance was measured at 517 nm using a UV visible spectrophotometer (UV-Visible spectrophotometer, Spec M02, Spectronic Camspec Ltd., Garforth, UK). The percentage radical scavenging ability was calculated using the following formula, and for each sample, 50% inhibitory concentration (IC50) was calculated. The experiment was repeated twice. Thus, the total number of replicates per temperature treatment per day was six.

$$\mathrm{Inhibition}\;(\%)=\frac{Absorbance\;control\left(nm\right)-Absorbance\;sample\left(nm\right)}{Absorbance\;control\left(nm\right)}\times 100$$

### 2.3. Sensory Evaluation

Sensory evaluation of stored microgreens was carried out according to the methods described by Berba & Uchanski [19] and Xiao et al. [30] with modifications. Four mustard microgreen bags were drawn at 2, 4, 7, and 14 days each from samples stored at 5 and 10 °C; 2, 4, and 7 days from samples stored at 15 °C; and 2 days from samples stored at 20 and 25 °C; these were used in the sensory evaluation test. Four bags, each with 15 g of freshly harvested microgreens, were used as the control. Microgreens from all four bags per temperature treatment per one storage duration were pooled and washed thoroughly with distilled water and subsequently with sodium chloride solution. Approximately 3–5 g of microgreens from each storage treatment (one replicate) were labeled with 3-digit random numbers and were randomly served to the panelists. Freshness, color, off-odor, sliminess, mustard flavor, off-flavor, and overall quality of microgreens were evaluated using a 5-point Descriptive Rating Scale (Table 1) on day 0, 2, 4, 7, and 14 after storage by a panel of fifteen members (3 males and 12 females; aged between 23 and 43 years). At the beginning of the evaluation, the panelists were made familiar with the evaluation procedure and the sensory attributes they were to evaluate. The panelists were given the opportunity to clarify any doubts about the sensory evaluation prior to its commencement.

The number of samples served during a day depended on the number of samples retrieved from different storage temperatures due to different rates of spoilage of microgreens at different storage temperatures. On the first day of sensory evaluation (day 0), fresh microgreens were evaluated by the panelists; on day 2, samples stored at 5, 10, 15, 20, and 25 °C; on day 4 and 7, samples stored at 5, 10, and 15 °C; and on day 14, samples stored at 5 and 10 °C. Between sample tasting, the panelists were instructed to wash their mouths with water to clean their palates. Since the evaluations were conducted for five days, during each evaluation day, a freshly harvested mustard microgreens sample was provided to the panelists as a reference and were not used in the calculations. The sensory evaluation was conducted once; thus, the total number of replicates was 15.

### 2.4. Statistical Analysis

Percentage data were normalized by arcsine transformation prior to statistical analysis. Data were analyzed using one-way ANOVA. Fisher’s least significant difference procedure was used to compare treatments (*p* = 0.05). All analyses were carried out using Minitab version 17.1.0 and the graphs were constructed using Sigma Plot ver.13.0.

## 3. Results

**Figure 1:**
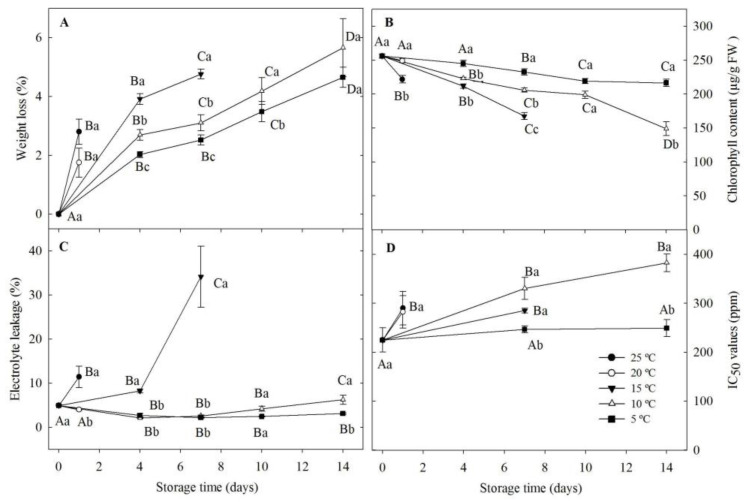
Effect of temperature on the changes in weight loss (**A**), total chlorophyll content (**B**), tissue electrolyte leakage (**C**), and antioxidant capacity (**D**) of mustard microgreens stored at different storage temperatures. Each data point value is expressed as mean ± SE; *n* = 8 for weight loss, total chlorophyll content, and tissue electrolyte leakage; and *n* = 3 for antioxidant capacity. Lowercase letters indicate significant differences (*p* < 0.05) between storage temperatures at a given storage duration, and uppercase letters indicate significant differences (*p* < 0.05) within different storage durations for a given storage temperature.

**Figure 2:**
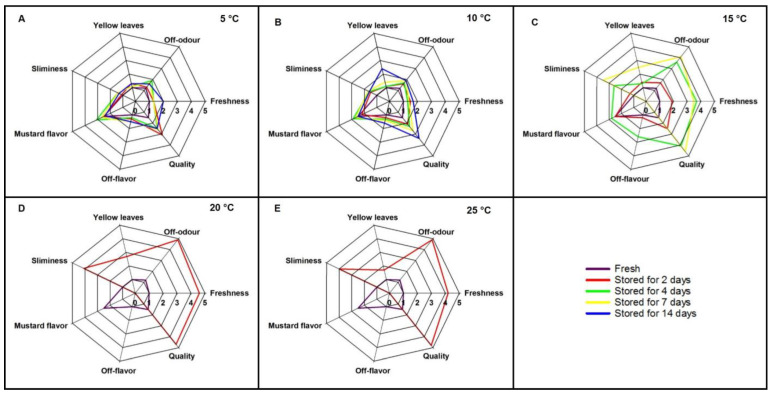
Spider diagrams of the sensory evaluation of mustard microgreens stored for 2, 4, 7, and 14 days (red, green, yellow, and blue lines, respectively) at different temperatures ((**A**) 5 °C; (**B**) 10 °C; (**C**) 15 °C; (**D**) 20 °C; and (**E**) 25 °C), compared to the fresh control (purple line) (*n* = 15).

The results revealed that the storage temperature significantly affected all the post harvest quality parameters measured in the study (Figure 1). As the temperature increased, the shelf life of microgreens decreased significantly (*p* < 0.05). Samples stored at 5 and 10 °C lasted 14 days under the respective storage temperatures. Based on the sensory analysis of the overall quality of microgreens, those stored for 14 days at 5 °C were identified as “good-fair” (Figure 2A) while the samples stored for 14 days at 10 °C were identified as “fair” (Figure 2B). Samples stored at 15 °C lasted for 7 days (Figure 2C), while those stored at 20 °C and 25 °C lasted only 1 day (Figure 2D,E).

### 3.1. Weight Loss

The weight loss increased with increasing storage temperature (Figure 1A). With storage time, a significant increase in weight loss was observed at all storage temperatures (*p* < 0.05). After one day of storage, a significant increase (*p* < 0.05) in weight loss was observed both at 20 °C and 25 °C (Figure 1A). On day 7, the percent weight loss at 5, 10, and 15 °C was 2.5, 3.10, and 4.76, respectively. On day 14, weight loss at 5 °C (4.65%) and 10 °C (5.65%) storage temperatures were not significantly different.

### 3.2. Total Chlorophyll Content

The initial total chlorophyll content of mustard leaves was approximately 250 µg/g FW (Figure 1B). Total chlorophyll content declined in all samples throughout storage. The highest chlorophyll retention was observed in samples held at 5 °C (216 µg/g FW) during the 14-day storage. In contrast, samples held at 10 and 15 °C retained total chlorophyll contents of 149 µg/g FW and 167 µg/g FW, respectively, on days 7 and 14. The samples kept at 20 °C for one day showed higher chlorophyll retention (249 µg/g) than the samples stored at 25 °C (221 µg/g FW).

### 3.3. Tissue Electrolyte Leakage

The initial electrolyte leakage was 4.98% (Figure 1C). When stored at 5 °C and 10 °C for 7 days, a significant reduction of 2.6% and 2.24%, respectively, in electrolyte leakage was observed during the first 7 days of storage. At the end of 14 days of storage at 10 °C, the electrolyte leakage significantly (*p* < 0.05) increased up to 6.27%. The highest electrolyte leakage (34%) was observed in samples kept at 15 °C on day 7. The electrolyte leakage significantly increased to 11% after one day of storage (*p* < 0.05) at 25 °C; however, no significant increase was observed at 20 °C after 1 day (4.04%). Compared to those stored at higher temperatures (15, 20, or 25 °C), samples stored at 5 °C and 10 °C showed significantly low electrolyte leakage (*p* < 0.05) on the final evaluation day. After 14 days of storage, samples stored at 5 °C showed the lowest electrolyte leakage (*p* < 0.05).

### 3.4. DPPH Radical Scavenging Antioxidant Activity

During the entire storage period, the highest antioxidant activity was exhibited by samples stored at 5 °C followed by 10, 15, 20, and 25 °C (Figure 1D, based on low IC50). Initial IC50 values of mustard microgreens were 228.27 ppm. There was no significant difference in antioxidant capacity during 14-day storage at 5 °C, and the initial antioxidant capacity was maintained throughout the storage period. Moreover, there was no significant difference between the antioxidant capacities of samples kept at 10 and 15 °C on day 7. The IC50 values significantly (*p* < 0.05) increased after 7 days of storage at 10 °C compared to initial values; however, they did not significantly change during the following seven days. The IC50 values of samples stored at 20 and 25 °C significantly increased after one day (*p* < 0.05).

### 3.5. Sensory Evaluation

The results of the descriptive rating scale of seven sensory attributes are shown in spider diagrams (Figure 2). All the sensory attributes of fresh microgreens were in the range of 1–2.5. After 14 days of storage, the sensory attributes of samples stored at 5 °C remained between 1 and 2.5 (Figure 2A). Yellow leaves and sliminess were not observed in samples stored at 5 °C during the entire storage period, and 25% of the samples were wilted by day 14, which maintained “good–fair” overall quality at the final evaluation. On day 2, samples stored at 5 °C reported a lower overall quality (“fair”) compared to day 14 (Figure 2A). Samples stored at 10 °C maintained “good-fair” overall quality for 4 days, and at the final evaluation, they showed 25% yellow leaves, 25% sliminess, and a “slight off odor”, and it had reached “fair-poor” overall quality (Figure 2B). The samples stored at 15 °C retained high sensory attributes for two days, and they deteriorated fast and became unacceptable after 7 days of storage (Figure 2C). When stored at 20 and 25 °C, all the sensory attributes degraded by day 2 and became inedible (Figure 2D,E). Therefore, the panelists were requested not to taste/evaluate the “mustard flavor” or “off-flavors” of the microgreens stored at 20 and 25 °C on day 2.

## 4. Discussion

Our results suggest that low-temperature storage alone is effective in prolonging the shelf life of highly perishable mustard microgreen. When compared to freshly harvested mustard microgreens, those stored in sealed 150 µm gauge polyethylene bags at 5 and 10 °C retained the same levels of tested postharvest and sensory attributes for 14 days. This observation suggests the importance of low-temperature storage to increase the shelf life of packaged mustard microgreens. According to the observed results, low-temperature conditions (5 and 10 °C) slowed down the spoilage of mustard microgreens more than the temperatures 15, 20, and 25 °C. Samples stored at 15 °C spoiled after 7 days, while those stored at 20 and 25 °C spoiled after 2 days of storage. Thus, weight loss, total chlorophyll content, electrolyte leakage, and IC50 values could not be evaluated beyond this period.

Temperature is the crucial factor that limits the shelf life of fresh produce [31,32]. The optimum storage temperature will mainly depend on the product type and cost. Thus, the determination of maximal storage temperature is important for retaining freshness, nutritional quality, and consumer acceptance. Among the postharvest attributes tested, weight loss of mustard microgreens was prominent with increasing storage temperature and duration. Similar results were reported previously for radish microgreens [16] and buckwheat microgreens [33]. Water loss is one of the major factors in the postharvest deterioration of fresh produce [34]. It results in quantitative losses (weight loss) and qualitative losses such as a decrease in the textural quality of the product. Microgreens are preferred among consumers for their fresh crispy texture [30]. Thus, water loss will directly affect the consumer acceptance of microgreens. Low-temperature storage of microgreens at 1–5 °C in LDPE bags with modified atmospheric conditions (high O_2_ and low CO_2_ concentrations), PET clamshell packaging, and postharvest treatments such as Ca, ClO_2_, citric acid wash, aloe vera gel treatments, etc., reduce the water loss and extend the shelf life of microgreens and maintain high consumer acceptance [2,22,33,34]. In our study, storage of mustard microgreens in 150 µm gauge polyethylene bags at 5 and 10 °C were effective in limiting the weight loss of microgreens to <6% during 14 days of storage. Thus, additional postharvest treatments are not required to extend the shelf life of mustard microgreens.

Tissue electrolyte leakage is an indicator of plant cell membrane weakening due to ripening or damage caused by physiological stress or mechanical injuries [35,36]. Electrolyte leakage leads to quality loss in fresh-cut produce during storage [37,38]. In this study, different storage temperatures have significantly affected tissue electrolyte leakage. The decrease in electrolyte leakage during the first four days at 5 and 10 °C was observed. Since the cut mustard microgreen was packed, high electrolyte leakage can occur in fresh samples on day 0. At low temperatures, the healing of cut surfaces may have contributed to the decline in electrolyte leakage [2]. Similar observations have been made for fresh-cut cilantro [36,37], buckwheat microgreens [2], broccoli microgreens [24], and radish microgreens [16]. The electrolyte leakage in mustard microgreens stored at 5 °C was minimal (<3%), while significantly increased electrolyte leakage was observed at 15 °C. Kou et al. [2] reported a similar pattern of low electrolyte leakage in buckwheat microgreens at 5 °C. However, when stored at 1 °C, buckwheat microgreens showed a significant increase in electrolyte leakage at 14 days of storage. Similarly, Ghoora & Srividya 2020 [34] reported a significantly high (11% and 16.3%) electrolyte leakage in Radish and Roselle microgreens, respectively, when stored at 5 °C in sealed LDPE bags for 8 days. Thus, it can be assumed that suboptimal storage of microgreens may cause chilling injuries which impair cell membrane function in stored microgreens, leading to increased electrolyte leakage. However, it should also be noted that the optimum storage temperature for radish microgreens is 1 °C, under which the quality attributes were retained for 28 days, with no chilling injuries [16]. Therefore, the optimum storage temperature of microgreens varies from species to species.

In this study, total chlorophyll content declined in all the stored samples, and the highest chlorophyll retention was observed in samples kept at 5 °C on all evaluation days. Xiao et al. [16] stated that the decrease in chlorophyll content depends on storage temperature. Lower temperatures result in greater chlorophyll retention than higher temperatures. The likely reason for this is the reduction in metabolic activities related to chlorophyll degradation under low temperatures [12]. The degradation of chlorophyll will cause the yellowing of microgreens, thus directly affecting consumer acceptance. Therefore, low-temperature storage is essential for the retention of chlorophyll. In the present study, the storage of microgreens under all tested temperatures, other than 5 °C, resulted in the yellowing of leaves. Therefore, the storage of mustard microgreens at 5 °C can be recommended for retaining high consumer acceptance.

When consumed regularly, species in the Brassicaceae family are known to reduce the health risk of cardiovascular diseases and several types of cancer [39]. These health-promoting benefits are due to the presence of antioxidant compounds such as allyl isothiocyanate, carotenoids, ascorbic acid, and flavonoids (isorhamnetin, kaempferol, quercetin, and isorhamnetin) [40]. Antioxidant compounds have the ability to protect cells against oxidative damage by directly scavenging free radicals and therefore could provide health benefits to humans [41]. Galani et al. [42] studied the effect of low-temperature storage (4 °C) for 15 days on the antioxidant activity of selected fruits and vegetables and reported that antioxidant activity significantly decreased with storage even at low temperatures. The IC50 values are calculated to determine the concentration of the antioxidant compounds required to inhibit 50% of free radicals. The lower the IC50 value, the higher the antioxidant capacity, and vice versa. In contrast to the results observed by Galani et al. [42], mustard microgreens stored at 5 °C in the current study retained the highest antioxidant capacity during 14 days of storage, and no decline in antioxidant activity was observed.

The sensory evaluation results indicated the effect of storage temperature on the sensory attributes of mustard microgreens. The sensory quality of mustard microgreens deteriorated at a high rate when stored at 15 °C or higher temperatures. When stored at 5 °C, a “good-fair” overall quality was observed after a 14-day storage period. However, a reduction in overall quality rating on day 2 was reported, and most probably, this may be due to the mishandling of the samples prior to serving. When considering all the sensory attributes, storing mustard microgreens in 150 µm polythene bags at 5 °C can be recommended for maintaining high consumer acceptance for 14 days. Similar results and conclusions were reported previously for radish microgreens by Xiao et al. [16]. Throughout the whole 14-day storage period, radish microgreens stored at 10 °C were rated highest in overall quality, followed by samples at 5 °C. Samples stored at 10 °C maintained acceptable visual quality until day 7 [16]. Berba & Uchanski [15] reported that when stored at 4 °C, the visual quality of arugula and red cabbage microgreens was retained for 14 days and 21 days, respectively. When stored at 10 °C, the shelf life of arugula and red cabbage microgreens was limited to 7 days and 14 days, respectively.

## 5. Conclusions

Storage temperature had a significant impact on all the postharvest quality parameters and sensory attributes. Compared to high storage temperatures of 10 °C or higher, storage at 5 °C resulted in a minimal loss in chlorophyll content, high antioxidant activity, and minimal tissue electrolyte leakage and retained high consumer acceptance within 14 days of storage. Therefore, storage of mustard microgreens in 150 µm polythene bags at 5 °C can be recommended.

## Figures and Tables

**Table 1:** Sensory attributes descriptive rating scale used in this study.

	Sensory Attributes						
Scale	Freshness	Color	Sliminess	Off Odor	Mustard Flavor	Off Flavor	Overall Quality
1	Product turgid	No yellow leaves	No sliminess	None	Very strong	None	Excellent
2	25% product wilted	25% yellow leaves	25% product slimy	Slight	Strong	Slight	Good
3	50% product wilted	50% yellow leaves	50% product slimy	Moderate	Moderate	Moderate	Fair
4	75% product wilted	75% yellow leaves	75% product slimy	Strong	Slight	Strong	Poor
5	100% product wilted	100% yellow leaves	100% product slimy	Very strong	None	Very strong	Very poor

## Data Availability

The data used to support the findings of this study are available from the corresponding author upon request.
